# Comparison of sociodemographic factors, lifestyle, and gastrointestinal symptoms between patients with endometriosis and IBS

**DOI:** 10.1186/s12876-025-04379-9

**Published:** 2025-10-21

**Authors:** Agnes Petersson, Bodil Roth, Ligita Jokubkiene, Povilas Sladkevicius, Bodil Ohlsson

**Affiliations:** 1 Department of Clinical Sciences, Lund University, Malmö, Sweden; 2 Department of Internal Medicine, Skåne University Hospital, Jan Waldenström street 15, floor 5, Malmö, 20502 Sweden; 3 Department of Obstetrics and Gynecology, Skåne University Hospital, Malmö, Sweden; 4 Obstetric, Gynaecological and Prenatal Ultrasound Research, Department of Clinical Sciences Malmo, Lund University, Malmö, Sweden

**Keywords:** Endometriosis, IBS, Gastrointestinal symptoms, Lifestyle habits, Sociodemographic factors, Trigger events

## Abstract

**Objectives:**

Endometriosis and Irritable bowel syndrome (IBS) are two common diseases with overlapping symptomatology, causing confusion and delay in the diagnostic process. The objective of this study was to identify differences between endometriosis and IBS diagnosed according to Rome IV, by comparing sociodemographic factors, lifestyle habits, and gastrointestinal symptoms.

**Materials and methods:**

Patients with endometriosis (*n* = 214), confirmed by laparoscopy or at transvaginal ultrasound, were recruited at the Department of Gynecology, Skåne University Hospital, Malmö. Patients with IBS (*n* = 199) were recruited from primary care centers, the Department of Gastroenterology, Skåne University Hospital, Malmö, and by advertisements at social media. All study participants answered questionnaires regarding sociodemographic factors, lifestyle habits, and medical history. Gastrointestinal symptoms were evaluated using the validated Visual Analog Scale for Irritable Bowel Syndrome (VAS-IBS).

**Results:**

There were limited differences in sociodemographic factors and lifestyle habits between women with endometriosis and IBS. However, endometriosis patients mainly needed analgetic treatment, opioids in 9.3% of cases, whereas IBS patients often needed drugs for intestinal dysfunction. GI symptoms were more aggravated in IBS than in endometriosis, especially diarrhea and constipation. The initial trigger events differed between the two diseases: menarche being most common in endometriosis and stress or infection/antibiotic treatment being most common in IBS. Both groups reported improvement of GI symptoms after dietary changes.

**Conclusions:**

The findings indicate that a thorough anamnesis about onset of disease and medication needs together with rating of gastrointestinal symptoms by validated instruments could be useful tools to differentiate between endometriosis and IBS in clinical practice.

## Introduction

 Endometriosis is a chronic inflammatory disease characterized by endometrial-like tissue outside the uterus [1]. The disease can be found in different locations and may present with varying symptoms such as pelvic pain, dysmenorrhea, and infertility [2]. The prevalence of endometriosis varies in different studies and depends on diagnostic methods. Transvaginal ultrasound found endometriosis in 25% of symptomatic women in reproductive age [3]. The disease is associated with irritable bowel syndrome (IBS) and mental illness in the form of depression, anxiety, and eating disorders [4–6]. Previously, the golden standard for endometriosis diagnosis was laparoscopy with histopathological confirmation [2, 7]. However, since 2022 the golden standard for diagnosis is transvaginal ultrasound [8].

IBS is a disease of the gut-brain interaction (DGBI), most frequently found in women, with a global prevalence of 1–25% and a pooled prevalence of 3.8% [9]. IBS is associated with several other diseases, e.g., depression, headache, and fibromyalgia [10]. The IBS diagnosis is symptom-based by using the Rome IV criteria [11].

According to meta-analyses, there is a two- to three-fold increased risk for endometriosis patients to also have IBS [5, 12], with a pooled IBS prevalence of 23.4% in endometriosis [12]. The etiology and pathology behind the diseases are unknown. Visceral hypersensitivity are parts of the pathophysiological mechanisms in both diseases [13, 14]. Inflammation with elevated pro-inflammatory cytokines is evident in endometriosis [15], but has not been possible to confirm in IBS although a low-grade inflammation has been proposed [16]. In a population-based study of both sexes, self-reported IBS according to Rome III criteria was associated with present and former smoking and inversely associated with alcohol intake compared with the general population [17]. The extended cohort only including women, showed that both endometriosis and IBS were associated with sick leave, endometriosis was positively associated with former smoking and inversely association with Body Mass Index (BMI), and IBS was associated with present smoking [4]. In a cross-sectional endometriosis study, the disease was inversely associated with alcohol, physical activity, and BMI compared to controls from the general population [18]. Comparison of gastrointestinal (GI) symptoms did not show any statistically significant differences between endometriosis and IBS in a population-based cohort [4]. However, when comparing endometriosis and IBS from two cohorts recruited at a hospital, IBS patients had more severe GI symptoms, except for constipation, and worse psychological well-being [19]. The prevalence of IBS is much lower according to the Rome IV criteria (3.8%) compared with the previous Rome III criteria (9.2%) [9], and no comparison is performed between endometriosis and patients diagnosed with IBS according to Rome IV [11].

Since there is no simple screening method to diagnose endometriosis, and since women with endometriosis may fulfill the Rome criteria [11], endometriosis is often misdiagnosed as IBS [20]. The diseases have quite different etiology and demand different treatments, which underlines the importance of correct diagnosis. Our hypothesis was that it should be possible to identify clinical differences between endometriosis and IBS. The aim of the present study was therefore to compare sociodemographic factors, lifestyle habits, and GI symptoms between endometriosis and IBS diagnosed according to Rome IV.

## Materials and methods

This study was approved by the Ethics Review Board of Lund University, 2012/564 and 2016/56 for endometriosis, and 2017/171, 2017/192, and 2021–05407-01 for IBS. All subjects gave their written, informed consent before inclusion.

### Study design

Patients with endometriosis (*n* = 214) or IBS (*n* = 199) were recruited at Skåne University Hospital, Malmö. All patients answered a questionnaire regarding clinical data and completed the validated Visual Analogue Scale for Irritable Bowel Syndrome (VAS-IBS) [21]. Comparisons in sociodemographic factors, lifestyle habits, and GI symptoms were performed between the groups.

## Endometriosis patients

Recruitment of endometriosis patients took place during March 2013–March 2017, and February 2022–March 2023 at the Department of Gynecology at Skåne University Hospital, Malmö, Sweden. Exclusion criteria were multiple or severe somatic or psychiatric comorbidities, and current pregnancy. During the first inclusion period, patients were identified in medical records using the International Classification of Diseases and Related Health Problems, ICD-10, N80, according to previously described criteria with laparoscopic-verified endometriosis [22]. Between 2013 and 2017, 605 patients were identified. Of those, 307 declined to participate, 72 had moved from the region, 32 had significant comorbidity, 18 had an uncertain diagnosis, and four denied the diagnosis, leaving 172 women included [18]. For this study, 32 women were excluded because of having a diagnosis of IBS, leaving 140 women analyzed for clinical data.

**Fig. 1:**
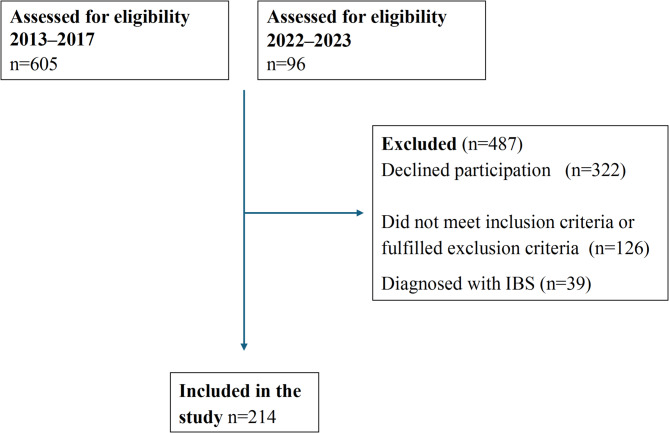
Flow chart of the inclusion and exclusion of patients with endometriosis. IBS, irritable bowel syndrome

During the second inclusion period, the method for diagnosis was changed due to updated guidelines [8]. Patients were systematically examined by experienced endometriosis ultrasound examiners according to recommendations from the International deep endometriosis analysis (IDEA) group [23]. Those who received a diagnosis of endometriosis confirmed by transvaginal ultrasonography were asked to participate in the study. Between 2022 and 2023, 96 patients fulfilled the inclusion criteria and were asked to participate in the study. Of those, 15 declined to participate. Seven women were excluded because of having a diagnosis of IBS, leaving 74 women to be included and analyzed for clinical data. Altogether, 214 patients with endometriosis were included (Fig. 1).

Localization of endometriosis lesions were divided into isolated ovarian lesions, or involvement of one or more of bowel, peritoneum, pouch of Douglas, rectovaginal septum, sacrouterine ligaments, urine bladder, or vesicouterine pouch.

### IBS patients

Recruitment of IBS patients took place during 2018–2019 and 2022–2023 from primary care centers, the Department of Gastroenterology at Skåne University Hospital, Malmö, and by advertisements on social media. The patients were recruited to participate in a dietary trial. Patients were identified from health care centers using the ICD-10, K58.0, K58.1, K58.2, K58.3, K58.8, and K58.9 and were contacted by email and telephone. During the first inclusion period, 697 patients were contacted. Of them, 145 were willing to participate. Later, 22 did not meet the inclusion criteria and 18 declined to participate, leaving 105 included. All men (*n* = 23) and one patient having a diagnosis of endometriosis were excluded from this study, leaving 81 women finally included [24].

**Fig. 2:**
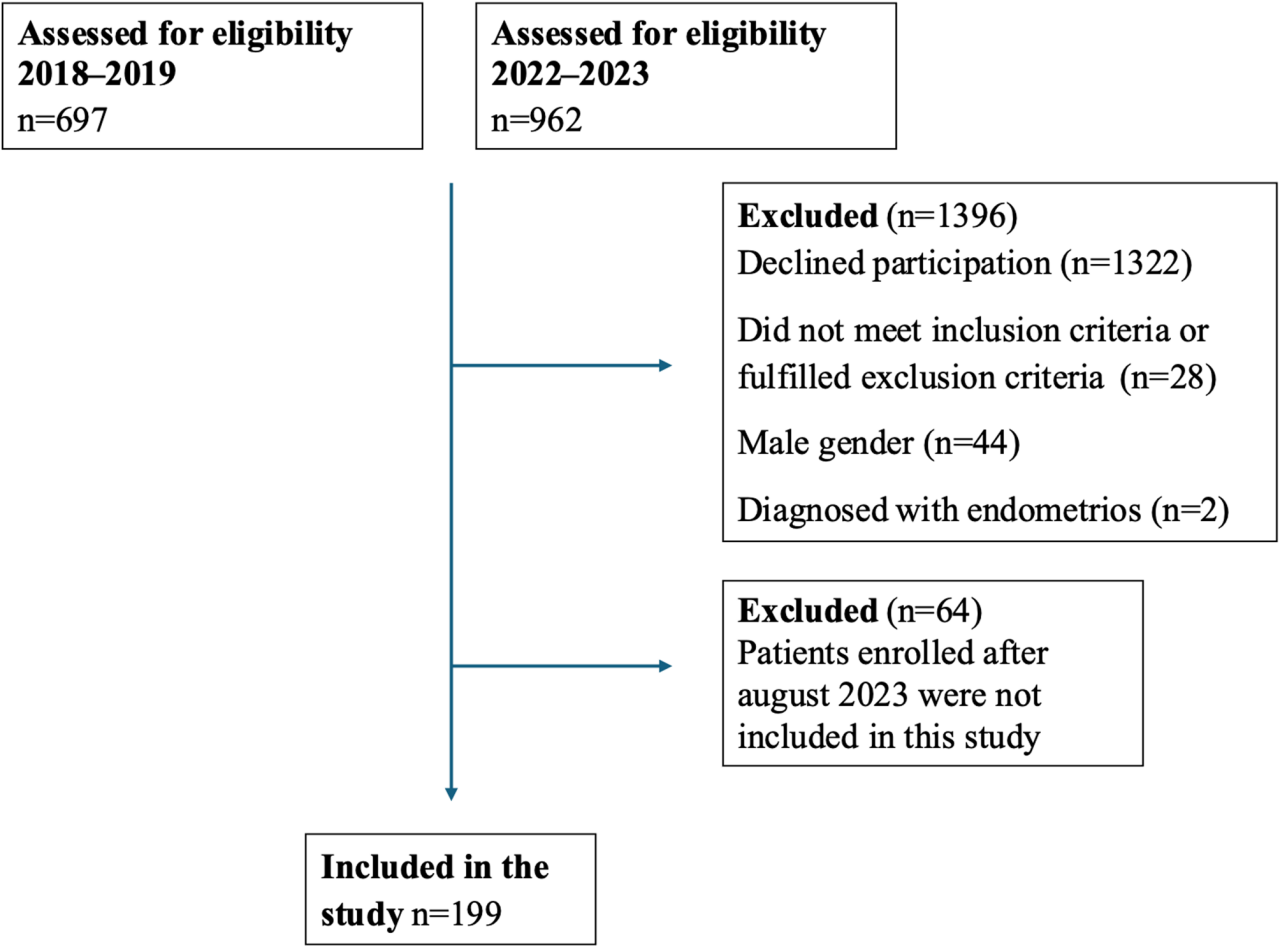
Flow chart of the inclusion and exclusion of patients with irritable bowel syndrome (IBS)

During the second inclusion period, 744 patients from the health care centers were contacted. Of them, 58 were willing to participate. From social media, 218 who had received an IBS diagnosis signed up for participation. Later, 6 did not meet the inclusion criteria and 66 declined to participate. All men (*n* = 21) and one patient having a diagnosis of endometriosis were excluded. Only patients included before August 2023 are covered in this study, leaving 118 finally included [25]. Altogether, 199 women with IBS were finally included in the analysis (Fig. 2). Celiac disease was excluded in all IBS patients by analysis of transglutaminase antibodies.

### Study questionnaires

All patients answered a previously developed questionnaire addressing sociodemographic factors, lifestyle habits, medical history, pharmacological treatments, and issues related to their diagnoses [18].

### Visual analog scale for irritable bowel disease

GI symptoms were estimated using the validated VAS-IBS, measuring abdominal pain, diarrhea, constipation, bloating and flatulence, vomiting and nausea, psychological well-being, and intestinal symptoms’ influence on daily life on scales from 0 to 100 mm, where 0 represents no symptoms and 100 represents severe symptoms. The item psychological well-being has been used together with the established questionnaires Experiences in Close Relationships (ECR-36), Rosenberg Self-Esteem Scale (RSES), and the Sense of Coherence (SOC-13), and the item was found to strongly correlate to positive and negative aspects of psychological well-being, anxiety in close relations, self-esteem, and coping skills [26]. The scales were inverted from the original format [21]. Reference values are available from healthy women [27].

### Rome IV questionnaire

All patients with IBS answered the Rome IV questionnaire, developed to diagnose DGBI [28]. Questions 40–48 in the Swedish version were used to diagnose and classify the IBS patients. License was obtained from the Rome Foundation, Inc. (Raleigh, NC, USA).

### Data categorization

BMI was categorized into < 25, 25–29.9, and ≥ 30 kg/m^2^ according to the World Health Organization (WHO) standard [29]. Education level was grouped into primary school, secondary school, or at least one year of university studies. Occupation was categorized into working, sick leave, retired, unemployed, and studying. Marital status was divided into living alone, married/partners living together, and other e.g., partners not living together or living with others than partner. Smoking was categorized into never smokers, former smokers, present irregular smokers, and regular smokers. Alcohol intake was divided into < 1 standard glass per week, 1–4 standard glasses per week, 5–9 standard glasses per week, and >10 standard glasses per week. Physical activity per week rendering breathlessness was categorized into never, < 30 min, 30–60 min, 60–90 min, 90–120 min, and > 120 min.

### Statistical analyses

The SPSS for Windows (version 28.0; IBM) statistical software package was used for statistical analyses. Fisher´s exact test was used to compare dichotomous variables in endometriosis and IBS. Binary logistic regression was used with endometriosis or IBS as dependent variable to estimate odds ratios (OR) and 95% confidence intervals (CI) for the independent variables age, BMI, education, occupation, marital status, smoking, alcohol, and physical activity. Adjusted ORs were calculated with all variables included. GI symptoms between groups were compared using generalized linear model, adjusted for age and occupation, since these parameters differed between groups in the adjusted logistic regression model. Data is presented as numbers (%), median (interquartile range [IQR]), and β or OR (95% CI). Missing data were excluded from analyses. *P* < 0.05 was considered statistically significant.

## Results

### Subject characteristics

Patients with endometriosis were younger than patients with IBS (*p* < 0.001) and were more seldom studying (*p* = 0.006). No significant differences were identified between the groups regarding education, marital status, smoking, alcohol consumption, or physical activity (Table 1). The most common localization of endometriosis was isolated ovarian endometriosis (Table 2).

**Table 1:** Patient characteristics

	Endo *N* = 214	IBS *N* = 199	Crude OR (95% CI)	*P*-value	Adjusted OR (95% CI)	*P*-value
Age (years)	38 (33–43)	43 (33–55)	1.06 (1.04–1.08)	**< 0.001**	1.06 (1.03–1.09)	**< 0.001**
BMI categories						
< 25	117 (54.9)	108 (54.3)	1 (reference)		1 (reference)	
25.0–29.9	63 (29.6)	60 (30,2)	1.02 (0.66–1.58)	0.945	0.98 (0.56–1.55)	0.777
≥ 30	27 (12.7)	27 (13.6)	1.08 (0.60–1.96)	0.792	1.16 (0.58–2.32)	0.684
Education *n* (%)						
* Missing value*	1					
Primary school	6 (2.8)	7 (3.5)	1 (reference)		1 (reference)	
Secondary school	43 (20.1)	32 (16.1)	0.64 (0.20–2.08)	0.456	0.52 (0.08–3.21)	0.479
Higher education	164 (76.6)	160 (80.4)	0.84 (0.28–2.54)	0.753	0.72 (0.12–4.20)	0.717
Occupation *n* (%)						
* Missing value*	1	2				
Working full time	128 (59.8)	111 (55.8)	1 (reference)		1 (reference)	
Working 51–99%	39 (18.2)	24 (12.1)	0.71 (0.40–1.25)	0.237	0.67 (0.35–1.26)	0.116
Working 1–50%	11 (5.1)	7 (3.5)	0.73 (0.28–1.96)	0.536	0.62 (0.23–1.69)	0.939
Sick leave	10 (4.7)	8 (4.0)	0.92 (0.35–2.42)	0.870	0.68 (0.24–1.94)	0.783
Retired	0	21 (10.6)	– (0.00–)	0.998	4.74 (0.85–26.55)	0.998
Unemployed	9 (4.2)	5 (2.5)	0.64 (0.21 − 1.97)	0.437	0.83 (0.25–2.76)	0.514
Studying	16 (7.5)	21 (10.6)	1.51 (0.75–3.04)	0.245	3.68 (1.58–8.58)	**0.006**
Marital status *n* (%)						
Married/cohabiting partner	149 (69.6)	129 (64.8)	1 (reference)		1 (reference)	
Single/living alone	54 (25.2)	61 (30.7)	0.77 (0.50–1.19)	0.231	0.91 (0.54–1.52)	0.712
Other	11 (5.1)	9 (4.5)	1.24 (0.28–1.88)	0.508	1.06 (0.36–3.13)	0.922
Smoking *n* (%)						
* Missing value*	1					
Never	136 (63.6)	104 (52.3)	1 (reference)		1 (reference)	
Former	43 (20.1)	71 (35.7)	0.80 (0.28–2.25)	0.658	0.81 (0.23–2.89)	0.742
Present, irregularly	19 (8.9)	12 (6.0)	2.06 (0.88–4.82)	0.094	1.60 (0.55–4.65)	0.391
Present, regularly	15 (7.0)	12 (6.0)	0.96 (0.43–2.13)	0.912	0.94 (0.34–2.63)	0.906
Alcohol intake per week, glasses *n* (%)						
* Missing value*	1					
< 1	134 (62.6)	95 (47.7)	1 (reference)		1 (reference)	
1–4	66 (30.8)	76 (38.2)	1.64 (1.07–2.50)	**0.022**	1.50 (0.91–2.46)	0.111
5–9	11 (5.1)	24 (12.1)	3.06 (1.43–6.54)	**0.004**	2.35 (0.99–5.58)	0.052
≥ 10	2 (1.0)	4 (2.0)	1.40 (0.28–7.09)	0.684	1.40 (0.22–8.85)	0.723
Physical activity per week, minutes *n* (%)						
* Missing value*	2					
Never	30 (14.0)	22 (11.1)	1 (reference)		1 (reference)	
< 30	41 (19.2)	36 (18.1)	1.20 (0.59–2.43)	0.619	1.03 (0.45–2.34)	0.954
30–60	38 (17.8)	35 (17.6)	1.26 (0.61–2.57)	0.533	1.11 (0.48–2.34)	0.802
60–90	37 (17.3)	31 (15.6)	1.14 (0.55–2.37)	0.720	0.97 (0.48–2.58)	0.952
90–120	23 (10.7)	25 (12.6)	1.48 (0.67–3.26)	0.329	0.85 (0.32–2.25)	0.735
> 120	43 (20.1)	50 (25.1)	1.59 (0.80–3.15)	0.187	1.74 (0.77–3.93)	0.184

Values are presented as median (interquartile range) or numbers (%). Binary logistic regression model was used to calculate crude and adjusted (age, BMI, education, occupation, marital status, smoking, alcohol and physical activity) odds ratio (OR) (95% confidence interval [CI]). *P* < 0.05 was considered statistically significant. Significant values are bold

*Endo* endometriosis, *IBS* irritable bowel syndrome, *BMI* body mass index - categories according to the WHO classification [28]

**Table 2:** Localization of endometriosis lesions

Localization	Number (%)
Isolated ovarian	75 (35.0)
Bowel	54 (25.2)
Peritoneum	24 (11.2)
Pouch of Douglas	43 (20.1)
Rectovaginal septum	2 (0.9)
Sacrouterine ligaments	56 (26.2)
Urine bladder	4 (1.9)
Vesicouterine pouch	14 (6.5)

Localization of endometriosis lesions identified by laparoscopy or transvaginal ultrasound. Multiple localizations are possible except in isolated ovarian

### Co-morbidity and pharmacological treatment

The most common present comorbidities in endometriosis were depression (*n* = 19, 8.9%), hypothyroidism (*n* = 18, 8.4%), and migraine (*n* = 15, 7.0%). In IBS, hypothyroidism (*n* = 18, 9.0%), asthma (*n* = 15, 7.5%), and migraine (*n* = 13, 6.5%) were most common, with significant differences between groups only in the prevalence of the burnout diagnosis. The prevalence of adenomyosis was 18 (24.3%) in the second cohort examined by ultrasound, which did not affect the degree of symptoms (data not shown). Patients with endometriosis used more hormonal treatment (37.9% vs. 20.1%) and analgetic drugs such as nonsteroidal anti-inflammatory drugs (NSAID) (18.7% vs. 9.0%) and opioids (9.3% vs. 0), whereas IBS patients used more drugs for the GI function such as proton pump inhibitors (PPI) (20.6% vs. 4.2%), laxatives (16.6% vs. 3.3%), and antidiarrheic drugs (7.5% vs. 1.4%) (Table 3).

**Table 3:** Current diagnoses and Pharmacological treatment in patients with endometriosis or irritable bowel syndrome (IBS)

	Endometriosis *N* = 214	IBS *N* = 199	*P*-value
Diseases *n* (%)			
Asthma	10 (4.7)	15 (7.5)	0.302
Allergy	23 (10.7)	28 (14.1)	0.114
Anxiety	13 (6.1)	6 (3.0)	0.163
Burnout	1 (0.5)	10 (5.0)	0.001
Depression	19 (8.9)	9 (4.5)	0.116
Fibromyalgia	4 (1.9)	9 (4.5)	0.055
Hypertension	9 (4.2)	12 (6.0)	0.503
Migraine	15 (7.0)	13 (6.5)	1.000
Psoriasis	2 (0.9)	2 (1.0)	0.232
Psychiatric disease	27 (12.6)	14 (7.0)	0.070
Rheumatoid diseases	4 (1.9)	3 (1.5)	0.675
Thyroid disease			
Normal function	193 (90.2)	179 (89.9)	1.000
Hypothyroidism	18 (8.4)	18 (9.0)	0.863
Hyperthyroidism	3 (1.4)	2 (1.0)	1.000
Drugs *n* (%)			
Allergy and asthma medicine	15 (7.0)	20 (10.1)	0.292
Hypertension medication	7 (3.3)	13 (6.5)	0.168
Laxatives and bulking agents	7 (3.3)	33 (16.6)	**< 0.001**
Levothyroxine	19 (8.9)	19 (9.5)	0.866
Loperamide	3 (1.4)	15 (7.5)	**0.003**
NSAID	40 (18.7)	18 (9.0)	**0.007**
Opioids	20 (9.3)	0	**< 0.001**
Paracetamol	28 (13.1)	27 (13.6)	0.886
PPI	9 (4.2)	41 (20.6)	**< 0.001**
SSRI/SNRI	34 (15.9)	23 (11.6)	0.253
Hormonal treatment	81 (37.9)	40 (20.1)	**< 0.001**
Combined estrogen-gestagen or estrogen only	39 (18.2)	26 (13.1)	0.177
Gestagens only	35 (16.4)	15 (7.5)	**0.007**
GnRH analogs	13 (6.1)	0	**< 0.001**

Results are shown as numbers (%). More than one drug could be used simultaneously. Fisher´s exact test. *P*<0.05 was considered statistically significant. Significant values are bold

*NSAID* nonsteroidal anti-inflammatory drugs, *PPI* proton pump inhibitors, *SNRI* serotonin and norepinephrine reuptake inhibitors, *SSRI* selective serotonin reuptake inhibitors

### Gastrointestinal symptoms

Patients with IBS were divided into subgroups where 38 (19.1%) patients had constipation-predominant IBS (IBS-D), 51 (25.6%) had diarrhea-predominant IBS (IBS-D), 73 (36.7%) had mixed IBS (IBS-M), 9 (4.5%) had unspecified IBS (IBS-U), and 27 (13.6%) had unspecified functional bowel disorder (FBD). Unspecified FBD includes patients who had weekly abdominal pain in association with diarrhea or constipation less than 30% of the time. Patients with IBS had more severe GI symptoms than endometriosis regarding abdominal pain (*p* < 0.001), diarrhea (*p* < 0.001), constipation (*p* < 0.001), bloating and flatulence (*p* < 0.001), vomiting and nausea (*p* < 0.042), intestinal symptoms´ influence on daily life (*p* < 0.001), and psychological well-being (*p* < 0.003), after adjustment for confounders (Table 4).

**Table 4:** Gastrointestinal symptoms in patients with endometriosis and irritable bowel syndrome (IBS)

	Endometriosis *N* = 214	IBS *N* = 199	β value (95% CI)	*P*-value
Abdominal pain	40 (9–72)	50 (34–65)	9.50 (3.74–15.27)	**< 0.001**
*Reference value*	5 (1–15)	5 (1–15)		
*Missing value*	3			
Diarrhea	11 (0–48)	52 (10–73)	23.03 (16.66–29.39)	**< 0.001**
*Reference value*	3 (0–10)	3 (0–10)		
Constipation	28 (0–65)	54 (10–75)	13.79 (7.05–20.54)	**< 0.001**
*Reference value*	9 (1–22)	9 (1–22)		
Bloating and flatulence	50 (15–76)	76 (62–88)	26.27 (20.4–32.12)	**< 0.001**
*Reference value*	14 (1–29)	14 (1–29)		
*Missing value*		1		
Vomiting and nausea	6 (0–35)	14 (2–40)	5.68 (0.22–11.14)	**0.042**
*Reference value*	2 (0–3)	2 (0–3)		
Intestinal symptoms´ influence on daily life	35 (5–77)	71 (57–83)	29.33 (23.39–35.27)	**< 0.001**
*Reference value*	2 (0–18)	2 (0–18)		
Psychological well-being	32 (6–62)	47 (20–64)	8.78 (3.07–14.50)	**0.003**
*Reference value*	4 (0–16)	4 (0–16)		

Gastrointestinal symptoms during the last 2 weeks were measured by the visual analog scale for irritable bowel syndrome (VAS-IBS) [21]. Reference values from healthy controls [27]. Generalized linear model was used to compare endometriosis and IBS, adjusted for age and occupation. Values are presented as median (interquartile range [IQR]) and β value (95% confidence interval [CI]). *P*-value < 0.05 was considered statistically significant. Significant values are bold

A total of 32 (15%) patients with endometriosis reported no GI symptoms on VAS-IBS. In endometriosis, 101 (47.2%) patients said that they were able to differentiate between abdominal pain from endometriosis or from the GI tract. Dietary changes due to GI symptoms had been tested by 51.6% of endometriosis patients and by 87.9% of IBS patients (*p* < 0.001). Of those, a total of 73.3% experienced improvement of symptoms after dietary changes in endometriosis compared to 72.0% in IBS (*p* = 1.000).

### Symptom debut and trigger factors

The median age for debut of GI symptoms in endometriosis was 24 years and in IBS 21 years. Median age for debut of endometriosis-related symptoms was 26 years. A total of 46 (21.5%) endometriosis patients and 54 (27.1%) IBS patients stated that there had been an initial trigger to their GI symptoms. Twenty-two (10.3%) endometriosis patients stated that menstruation triggered their symptoms debut compared with only one (0.5%) IBS patient (*p* < 0.001). The major triggering factor in IBS was a period with high stress level, which was reported by 25 (12.6%) patients compared with only five (2.3%) endometriosis patients (*p* < 0.001). In 15 (7.5%) IBS patients, debut of GI symptoms was triggered by an infection or antibiotic treatment, which was not reported by any with endometriosis (*p* < 0.001) (Table 5).

**Table 5:** Initial trigger factors to Gastrointestinal symptoms in endometriosis and irritable bowel syndrome (IBS)

	Endometriosis *N* = 214	IBS *N* = 199	*p*-value
Bowel surgery/ileus	3 (1.4)	0	0.249
Ended hormonal treatment	5 (2.3)	0	0.062
Infection/antibiotic treatment	0	15 (7.5)	**< 0.001**
Menarche/menstruation	22 (10.3)	1 (0.5)	**< 0.001**
Stressful life events	5 (2.3)	25 (12.6)	**< 0.001**
Others	11 (5.1)	13 (6.5)	0.576

Values are presented as numbers (%). Fisher´s exact test. *P*<0.05 was considered statistically significant. Significant values are bold

## Discussion

The main findings of this study were that differences in sociodemographic and lifestyle habits between patients with endometriosis and IBS were limited, but GI symptoms were more aggravated in IBS than in endometriosis. The initial trigger events differed between the two diseases, with menarche being the most common trigger in endometriosis and stress or infection/antibiotic treatment were the most common triggers in IBS. Endometriosis patients mainly needed analgetic treatment, whereas IBS patients more often needed drugs for intestinal dysfunction. Both groups reported improvement of GI symptoms after dietary changes.

The lower age in endometriosis may be explained by the recruitment route. During the second inclusion period for endometriosis, patients were included at the time they were diagnosed by ultrasonography. For IBS and the first endometriosis cohort, patients were included regardless of disease duration. Patients with endometriosis enrolled in this study were all recruited at a tertiary center while IBS were recruited from either primary care centers, a tertiary care center or actively signed up after advertisement for a dietary trial. Further, the first patients with endometriosis were recruited 2013 and the first patients with IBS were recruited 2018, whereas the subsequent patients were recruited during the same period, independently of disease. Nevertheless, differences in recruitment process and time may include a bias in the results.

In the present study where patients with coincident diagnoses were excluded, patients with IBS had more severe GI symptoms than patients with endometriosis with significant differences regarding abdominal pain, diarrhea, constipation, bloating and flatulence, vomiting and nausea, intestinal symptoms´ influence on daily life, and psychological well-being. This is in line with former research from other study cohorts [19], although the differences did not reach statistical significance in a population-based cohort with very few registered endometriosis cases and self-reported IBS [4]. This points to the importance of using validated questionnaires in daily practice instead of only verbal description of diffuse symptoms. The findings of low level of constipation, diarrhea, and the influence of symptoms on daily life, suggest endometriosis rather than IBS, which is difficult to discover without a questionnaire [30].

The VAS-IBS questionnaire reflects symptoms over the last 2 weeks [26]. Pain symptoms in endometriosis are known to vary between patients with different levels of chronicity and variation over the menstrual cycle [31]. Deep endometriosis that invades into the bowel can lead to symptoms such as painful bowel movement at time of menstruation [32]. In this study, 37.9% of patients with endometriosis were currently using systemic hormonal treatment. Thereby, they already had one of the first-line treatments for endometriosis-related symptoms, rendering loss of or less menstruation, which we can assume was affecting the results. We do not know the phase of the menstrual cycle in the patients when estimating their symptoms on VAS-IBS. Thus, the results may not reflect the more intense pain periods during the cycle. In similarity, symptom fluctuations in patients with IBS during the time of menstruation were not recognized [33].

Although IBS patients had more severe abdominal pain, endometriosis patients were much more often treated with analgetic drugs. This may be due to on-demand treatment around the menstrual phase. The high prevalence of opioid prescription in endometriosis must still be questioned since it seems to be of no benefit for them [34]. On the contrary, opioid prescription is contradictory in IBS since it aggravates abdominal pain and GI dysfunction [35]. During the last years, the problem with opioid treatment of chronic non-cancer pain (CNCP) has been more recognized and debated. Evidence supporting long-term benefits of chronic opioid use in CNCP is sparse, but associated harms of opioid-related mortality and risks of dependence, addiction, and abuse are well documented [36, 37]. Therefore, the prescription of opioids in endometriosis should be reduced, and replaced by other evidence-based treatments [38].

Since the two diseases endometriosis and IBS have quite different consequences and treatments for the women, it is important to set the correct diagnosis. Further, misdiagnosis in cohorts and populations obscures the picture of the diseases and may deteriorate the research. By taking a thorough anamnesis, it should be possible to separate those patients with GI symptoms who would be of most benefit to exclude endometriosis. Patients with inflammatory bowel disease (IBD) also have a high prevalence of concomitant endometriosis [39]. Thus, endometriosis should be excluded in patients with IBD and abdominal pain, to prevent unnecessary, inappropriate overtreatment of the patients. Apart from fluctuating pain intensity over the menstrual cycle, patients with a debut of GI symptoms at the time point of menarche must be thoroughly examined to exclude endometriosis. While pain fluctuation over the menstrual cycle is suggestive of endometriosis, it is however also shown in IBS [33, 40]. A history of GI infection and/or antibiotic treatment suggesting a post-infectious disease is almost pathognomonic for IBS [41]. Furthermore, stress as a triggering factor is also typical for IBS [42, 43]. Those who suffer from severe constipation and/or diarrhea are most probably susceptible to have IBS.

Both patients with endometriosis and IBS had benefits of dietary changes. The explanation may be that poor dietary habits are common in the society [44], and restriction in diets are useful for the majority [45]. The effect of improvements after a diet is difficult to use for discriminations between diseases or mechanisms, also because of high placebo effects in these conditions [46, 47].

A strength of this study is the clear diagnosis of endometriosis performed either by laparoscopy or systematic ultrasound examinations performed by experienced ultrasound examiners according to IDEA group recommendations [22]. There are several limitations of the study. One clear limitation is that patients with IBS potentially could have undiagnosed endometriosis. Endometriosis can be asymptomatic or present with diffuse symptoms, and although some of the women with IBS had been considered to suffer from endometriosis, all were not examined by ultrasound to exclude disease [8]. The completion of VAS-IBS was not controlled in relation to the menstrual cycle. This may have affected the symptom burden in IBS and diluted the symptom burden in endometriosis [33, 40]. Considering the high prevalence of anxiety and depression amongst these patients, a questionnaire such as quality of life (SF-36), Hospital Anxiety and Depression scale (HAD), or Patient Health Questionnaire-9 (PHQ-9) could have added more information about the psychological well-being and the self-reported medical history in addition to VAS-IBS. The study questionnaire did not include any questions about sexual health and fertility status, which is another limitation. Furthermore, some differences in time and processes of recruitment as well as recall accuracy regarding age for debut of symptoms and trigger factors are potential limitations.

### Conclusion

The anamnesis remains an essential element in the management of endometriosis and IBS. When estimated with a self-rating questionnaire, GI symptoms and abdominal pain were shown to be more aggravated in IBS than in endometriosis. There were also distinct differences in trigger events of symptom development between the diseases. The findings indicate that a thorough anamnesis combined with rating of symptoms with a validated questionnaire are important tools in clinical practice, to select which women who should be further examined to exclude endometriosis.

## Data Availability

The datasets used during the current study are available from the corresponding author on reasonable request.

## Abbreviations

BMI: Body mass index
CI: Confidence interval
DGBI: Disease of the gut-brain interaction
GI: Gastrointestinal
IDEA: International deep endometriosis analysis
IBS: Irritable bowel syndrome
NSAID: Nonsteroidal anti-inflammatory drugs
OR: Odds ratio
PPI: Proton pump inhibitors
SNRI: Serotonin and norepinephrine reuptake inhibitors
SSRI: Selective serotonin reuptake inhibitors
VAS-IBS: Visual analog scale for irritable bowel syndrome
WHO: World health organization
